# Aging-associated patterns in the expression of human endogenous retroviruses

**DOI:** 10.1371/journal.pone.0207407

**Published:** 2018-12-04

**Authors:** Tapio Nevalainen, Arttu Autio, Binisha Hamal Mishra, Saara Marttila, Marja Jylhä, Mikko Hurme

**Affiliations:** 1 Faculty of Medicine and Life Sciences, University of Tampere, Tampere, Finland; 2 Gerontology Research Center (GEREC), University of Tampere, Tampere, Finland; 3 Faculty of Social Sciences, University of Tampere, Tampere, Finland; Universitat des Saarlandes, GERMANY

## Abstract

Human endogenous retroviruses (HERV) are relics of ancient retroviral infections in our genome. Most of them have lost their coding capacity, but proviral RNA or protein have been observed in several disease states (e.g. in inflammatory and autoimmune diseases and malignancies). However, their clinical significance as well as their mechanisms of action have still remained elusive. As human aging is associated with several biological characteristics of these diseases, we now analyzed the aging-associated expression of the individual proviruses of two HERV families, HERV-K (91 proviruses) and HERV-W (213 proviruses) using genome-wide RNA-sequencing (RNA-seq). RNA was purified from blood cells derived from healthy young individuals (n = 7) and from nonagenarians (n = 7). The data indicated that in the case of HERV-K (HML-2) 33 proviruses had a detectable expression but in only 3 of those the expression levels were significantly different between the young and old individuals. In the HERV-W family expression was observed in 45 loci and only in one case the young/old difference was significant. However, applying hierarchical clustering on the HERV expression data resulted in the formation of two distinct clusters, one containing the young individuals and another the nonagenarians. This suggests, that even though the aging-associated differences in the expression levels of individual proviruses are minor, there seems to be some underlying aging-related pattern. These data indicate that aging does not have a strong effect on the expression of individual HERV proviruses, but instead several proviruses are affected moderately, leading to age-dependent expression profiles.

## Introduction

During mammalian evolution, integration of retroviral RNA into a germ line cell may have led into a formation of a provirus that is transmitted vertically and inherited in a Mendelian manner. In humans, these endogenous retroviruses (HERV) comprise ca. 8% of our genome [1]. While it is known that some retroelements of the human genome are still capable of retrotransposition, DNA sequences of the HERVs have accumulated mutations to the point where retrotransposition or formation of viral particles is not taking place anymore [2]. Despite this mutation-driven functional inactivation, there are hundreds of publications demonstrating associations between HERV expression and various disease states (malignancies, infections, neurological and autoimmune diseases), however, the causal relationship has remained enigmatic [3–6].

Since the mechanism of action cannot be explained by *de novo* insertional mutagenesis nor with the formation of viral particles, it has been proposed, that potential pathogenicity of the HERVs could simply underlie in the presence of proviral DNA, acting as a transcriptional regulatory sequence, modifying the expression of neighboring and even more distant genes. HERVs can do this for example by acting as transcription factor binding sites. From this hypothesis it naturally follows, that potential effects of the HERVs would be restricted in some genomic window around the primary proviral insertion site. However, there is also evidence supporting more global mode of action as HERVs have been shown to activate immune and inflammatory responses of the body directly. For example, their RNA could be recognized as a pathogen-associated molecular pattern (PAMP) by Toll-like receptors and this would induce type I interferon production contributing to the pathogenesis of autoinflammatory diseases [7]. Some HERVs are still able to encode an intact envelope protein (Env) and its presence has been observed in some viral infections or in autoimmune diseases [3–6]. It has been proposed that the mechanism of action of Env is based on the antigenicity of the molecule, possibly causing a polyclonal activation of lymphocytes, i.e. functioning as a “superantigen” [8].

As the diseases, where HERV-associations have been observed, demonstrate some of the fundamental and characteristic aspects of aging, e.g. increased level of inflammation and changes in the proportions of the various lymphocyte subsets [9,10], we now quantitated the RNA levels of all previously characterized proviruses of HERV-K (HML-2) and HERV-W families in peripheral blood mononuclear cells (PBMC) derived from young and 90-year old individuals. Aging-associated increase in the expression of several HERV families has been reported previously using quantitative PCR [11]. However, qPCR approach utilizes degenerate primers for each HERV family, thus missing the information regarding individual proviruses. RNA-sequencing possesses the capability to obtain this crucial data and hence it was the method of choice.

The most recent entrants to our genome are represented by HERV-K (HML-2) family (ca. 0.2–2 million years ago), of which Subramanian et al. have identified 91 full-length proviral sequences [12]. HERV-W represents an older group of HERVs (primary infection ca. 40 million years ago) and it contains 213 full-length or near full-length elements [13].

## Methods

### Study populations

Two populations, representing young and elderly individuals, were used. The young ones consisted of healthy laboratory personnel, all female, aged 26 to 32 years (n = 7, median age 28) who did not have any medically diagnosed chronic illnesses, were non-smokers and had not had any infections or received any vaccinations within the two weeks prior to blood sample collection. The elderly individuals (n = 7) were selected among relatively healthy, community living, non-frail, nonagenarian females, without any severe aging-associated diseases, that were participants in The Vitality 90+ study. The nonagenarians were born in 1920 and the samples were collected in 2014. The recruitment and characterization of participants were performed as has been reported previously [14]. The study participants provided their written informed consent. This study was conducted according to the principles expressed in the declaration of Helsinki, and the study protocol was approved by the ethics committee of the city of Tampere (1592/403/1996).

### Sample collection

Blood samples were collected by a trained laboratory technician in the laboratory facilities. All blood samples were drawn between 8 am and 12 am and collected into EDTA containing tubes. Samples were directly subjected to leucocyte separation on a Ficoll-Paque density gradient (Ficoll-Paque Premium, cat. no. 17-5442-03, GE Healthcare Bio-Sciences AB, Uppsala, Sweden). The PBMC layer was collected and cells used for RNA extraction were suspended in 150 μl of RNAlater solution (Ambion Inc., Austin, TX, USA). Nonagenarian and control samples were collected at the same time.

### RNA extraction

RNA used for RNA sequencing was purified using a miRNeasy mini kit (Qiagen, CA, USA) and the RNA used for PCR analysis using RNeasy mini kit (Qiagen, CA, USA) according to manufacturer’s protocol with on-column DNA digestion (Qiagen). The concentration and quality of the RNA was assessed with a NanoDrop ND-1000 spectrophotometer (NanoDrop Technologies, Wilmington, DE, USA).

### RNA sequencing

Agilent Bioanalyzer RNA nano chips (Agilent) were used to evaluate the integrity of total RNA and Qubit RNA–kit (Life Technologies) to quantitate RNA in samples. 1 μg of total RNA was used for ScriptSeq Complete Gold System (Epicentre) to ribodeplete rRNA and further for RNA-seq library preparation. SPRI beads (Agencourt AMPure XP, Beckman Coulter) were used for purification of RNAseq libraries. The library QC was evaluated on High Sensitivity chips by Agilent Bioanalyzer (Agilent). Paired-end sequencing of RNAseq libraries was done using Illumina HiSeq technology with a minimum of 60 million 2x100bp paired-end reads per sample.

### Data preprocessing and analysis

Raw reads were aligned to human genome reference build hg19 using TopHat v2.0.13 [15] with the default parameters. Only uniquely mapped reads were considered in the transcript abundance estimation and to this end SAMtools [16] was used to filter out reads mapping to multiple regions of the genome. The downstream analyses were all conducted using the tools in cufflinks2 v. 2.2.1 [17, 18]. The raw expression estimates were calculated using cuffquant and the expression were normalized using cuffnorm, which gives the normalized read counts and the fragments per kilobase per million values (FPKM) for each gene as an output. The geometric normalization method was used which scales the read counts as well as the FPKM values according to procedure described in [19].

The annotation data for HERV-K (HML-2) was from Subramanian et al. [12] and that for HERV-W from Grandi et al [13]. To ensure the robustness of the normalization the expressions of HERV elements were quantified and normalized together with ENSEMBL v. 82 gene reference set [20, 21]. For each individual study subject, a given HERV element was considered significantly expressed when the individual expression level exceeded normalized read count of 16 [22].

### Cluster analysis

Hierarchical clustering of the samples based on normalized read counts was done separately for both HERV-K (HML-2) and HERV-W. Spearman correlation was used as the distance metric, which is robust against outliers and non-Gaussian distributions, and can capture nonlinear relationships [23, 24]. Ward's minimum increase of sum-of-squares was used as the linkage method, which has been reported to perform better with gene expression data than the more traditional methods of average and complete linkage [23]. Multistep-multiscale bootstrap resampling was done to evaluate the uncertainty involved in the clustering [25]. Thousands of samples of varying sizes are randomly created from the data and then clustered. An approximately unbiased (AU) p-value is obtained, which indicates the bias corrected percentage of dendrogram variants where the specific cluster was observed.

## Results

The results of the RNA-seq analysis indicated that 33 HERV-K (HML-2) loci out of 91 had a detectable expression, but often at low level and not in all individuals. The expression levels of PBMCs derived from young and elderly individuals were generally similar. Only at three loci (1q22, 10p14 and 12q24.33) the difference was statistically significant as shown in Table 1.

**Table 1:** Median expression levels (normalized read counts) of HERV-K (HML-2) proviruses. Proviruses were deemed expressed if exhibiting a read count of 16 or more [22]. Known aliases are derived from Subramanian et al. [12].

HERV-K locus	Aliases	Median expression level (normalized read count) in nonagenarians/young controls		Number of nonagenarians/young controls expressing the provirus
1p31.1a	K4, K116, ERVK-1	6.99	6.30	2 / 1
1q21.3	-	7.76	18.72	1 / 5
**1q22**	**K102, K(C1b),K50a,ERVK-7**	**339.70**	261.01*	7 / 7
1q23.3	K110, K18,K(C1a), ERVK-18	95.31	78.79	7 / 7
1q32.2	-	39.31	42.30	7 / 7
3q12.3	K(II), ERVK-5	774.61	916.86	7 / 7
3q13.2	K106, K(C3),K68, ERVK-3	19.77	11.47	4 / 3
3q21.2	K(I), ERVK-4	10.81	19.72	2 / 7
4p16.1a	K17b	24.46	26.10	6 / 6
4p16.1b	-	15.61	9.44	3 / 1
4p16.3a	-	15.45	16.20	3 / 4
7q34	K(OLDAC004979),ERVK-15	66.42	74.16	7 / 7
8p23.1a	K115, ERVK-8	28.81	11.39	7 / 1
8p23.1b	K27	14.32	17.09	3 / 4
8p23.1c	-	13.13	22.86	3 / 6
9q34.11	K31	40.53	36.90	6 / 7
9q34.3	K30	1.01	3.81	0 / 1
**10p14**	**K(C11a), K33,ERVK-16**	**70.77**	18.00*	7 / 4
10q24.2	ERVK-17, c10_B	7.16	8.95	0 / 1
11p15.4	K7	7.29	12.35	0 / 3
11q12.1	-	8.19	14.65	3 / 3
11q12.3	K(OLDAC004127)	13.53	8.93	2 / 3
12p11.1	K50e	0.00	0.00	1 / 0
12q24.11	-	12.49	4.88	3 / 2
**12q24.33**	**-**	**87.55**	97.25*	7 / 7
14q11.2	-	54.12	27.67	7 / 7
16p13.3	-	2.62	4.41	0 / 1
19q11	K(C19), ERVK-19	2.31	4.91	0 / 1
19q13.12a	-	7.76	18.10	1 / 4
19q13.12b	K(OLDAC012309),KOLD12309	122.78	146.49	7 / 7
19q13.41	-	12.23	9.71	2 / 2
20q11.22	K(OLDAL136419),K59	13.17	8.57	2 / 2
22q11.21	K101, K(C22),ERVK-24	5.12	4.76	0 / 1

^*^ Statistically significant (Mann-Whitney U-test) differential expression and expressed in majority of samples.

In the case of HERV-W, the results were similar, in 45 proviruses out of 213 the read count was >16 at least in one individual, and in the case of Xp11.21 the difference in expression levels between the young and old was significant as shown in Table 2.

**Table 2:** Median expression levels (normalized read counts) of HERV-W proviruses. Proviruses were deemed expressed if exhibiting a read count of 16 or more [22].

HERV-W locus	Median expression level (normalized read count) in nonagenarians/young controls		Number of nonagenarians/young controls expressing the provirus
1p12	5.22	10.59	0 / 1
1p22.2a	19.16	26.36	6 / 7
1p34.2	57.92	48.72	7 / 7
1q22	18.79	11.82	6 / 3
1q32.1	8.35	11.82	1 / 3
1q42.13	36.67	33.35	7 / 7
2p16.2	86.88	89.99	7 / 7
2p23.1a	11.07	16.17	2 / 4
2q11.2	33.96	27.27	7 / 6
2q22.2	108.33	113.64	7 / 7
2q24.3	3.71	14.38	0 / 2
2q31.2a	52.18	31.76	6 / 5
2q32.3	7.24	13.64	1 / 3
3q11.2	5.31	6.67	1 / 1
3q13.31	169.19	182.88	7 / 7
3q13.32	53.11	58.60	7 / 7
3q23b	86.98	73.15	7 / 7
3q26.32	8.35	12.71	2 / 2
4p16.3	13.66	11.72	3 / 3
4q21.22	9.31	15.45	1 / 3
5q22.2	3.03	4.49	0 / 1
6p22.3	25.05	30.80	4 / 5
6q21a	132.84	111.17	7 / 7
6q21c	8.92	38.13	3 / 7
6q24.2a	8.71	10.00	0 / 1
6q27b	35.17	36.58	7 / 6
7p14.2	4.16	0.48	2 / 0
7q21.2	18.18	17.77	4 / 5
7q31.1a	0.00	1.93	0 / 1
8q21.11	10.34	9.18	0 / 1
9p13.3	17.74	16.31	4 / 4
10q24.1	26.89	25.16	7 / 5
11q14.1	34.83	36.36	7 / 7
11q14.2	15.86	4.69	3 / 0
12q24.31	37.57	37.27	6 / 7
13q13.3	16.55	13.64	4 / 1
14q21.2	29.71	31.63	6 / 7
14q32.11	9.39	7.06	1 / 0
15q21.3	14.61	8.89	2 / 2
17q12a	11.32	10.00	1 / 2
17q12b	8.35	9.09	1 / 2
17q22	29.22	25.42	6 / 6
18p11.31	9.90	9.63	2 / 0
19q13.2a	9.90	17.05	0 / 5
**Xp11.21**	**16.54**	34.60*	4 / 7

*Statistically significant (Mann-Whitney U-test) differential expression and expressed in majority of samples.

**Fig 1:**
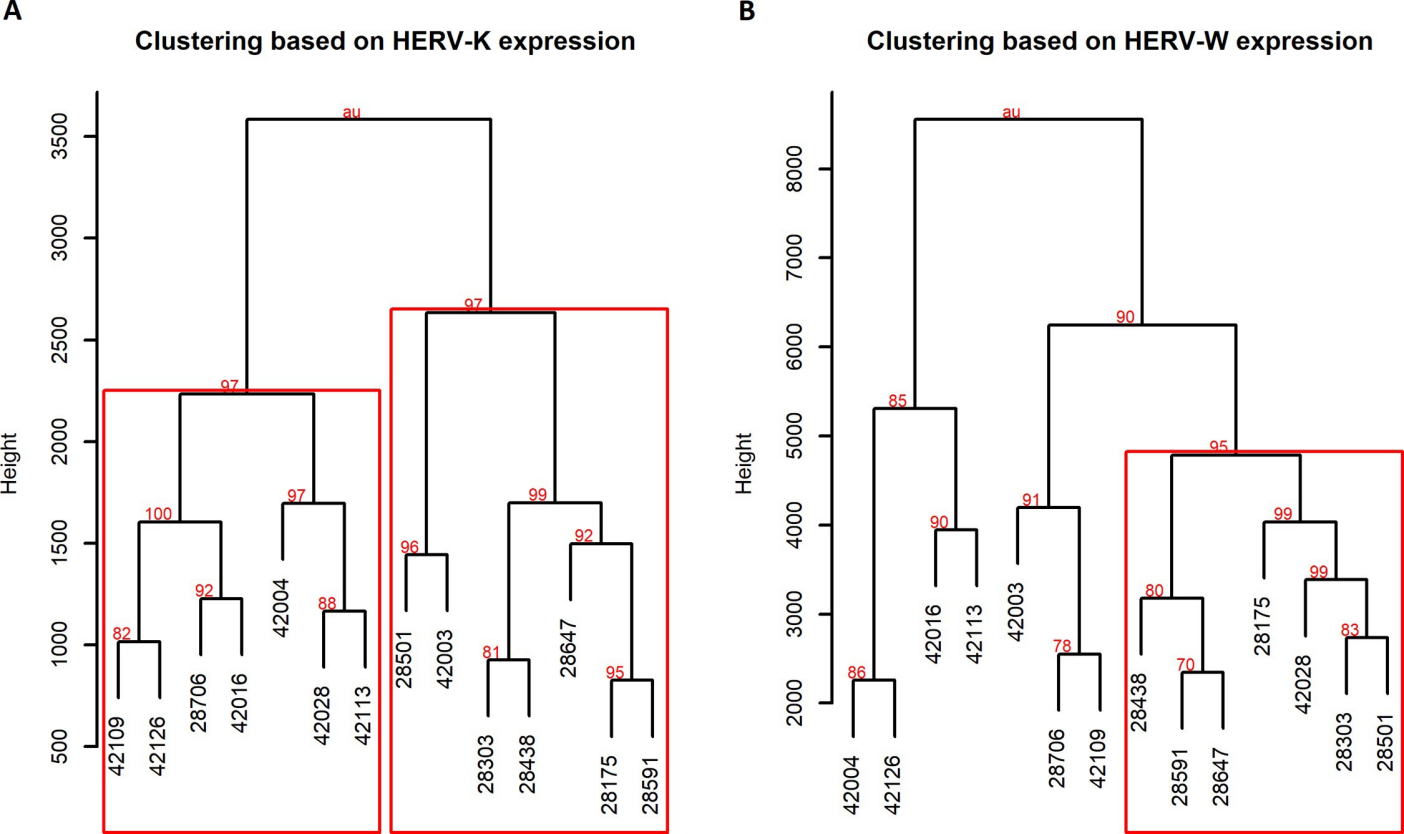
Hierarchical clustering of HERV-K(HML-2) and HERV-W proviruses. Hierarchical clustering of the samples was carried out with normalized (A) HERV-K (HML-2) and (B) HERV-W read counts, using Spearman correlation distance metric. Nonagenarian samples are indicated by an identifier starting with the number 2, while control sample identifiers start with 4. The height separating clusters has been calculated with Ward’s minimum increase of sum-of-squares linkage method and indicates proportional dissimilarity between clusters. The red squares indicate clusters that were deemed statistically significant through bootstrap resampling. AU p-value, in red font, indicates the bias corrected percentage of dendrogram variants where the cluster was present.

**Fig 2:**
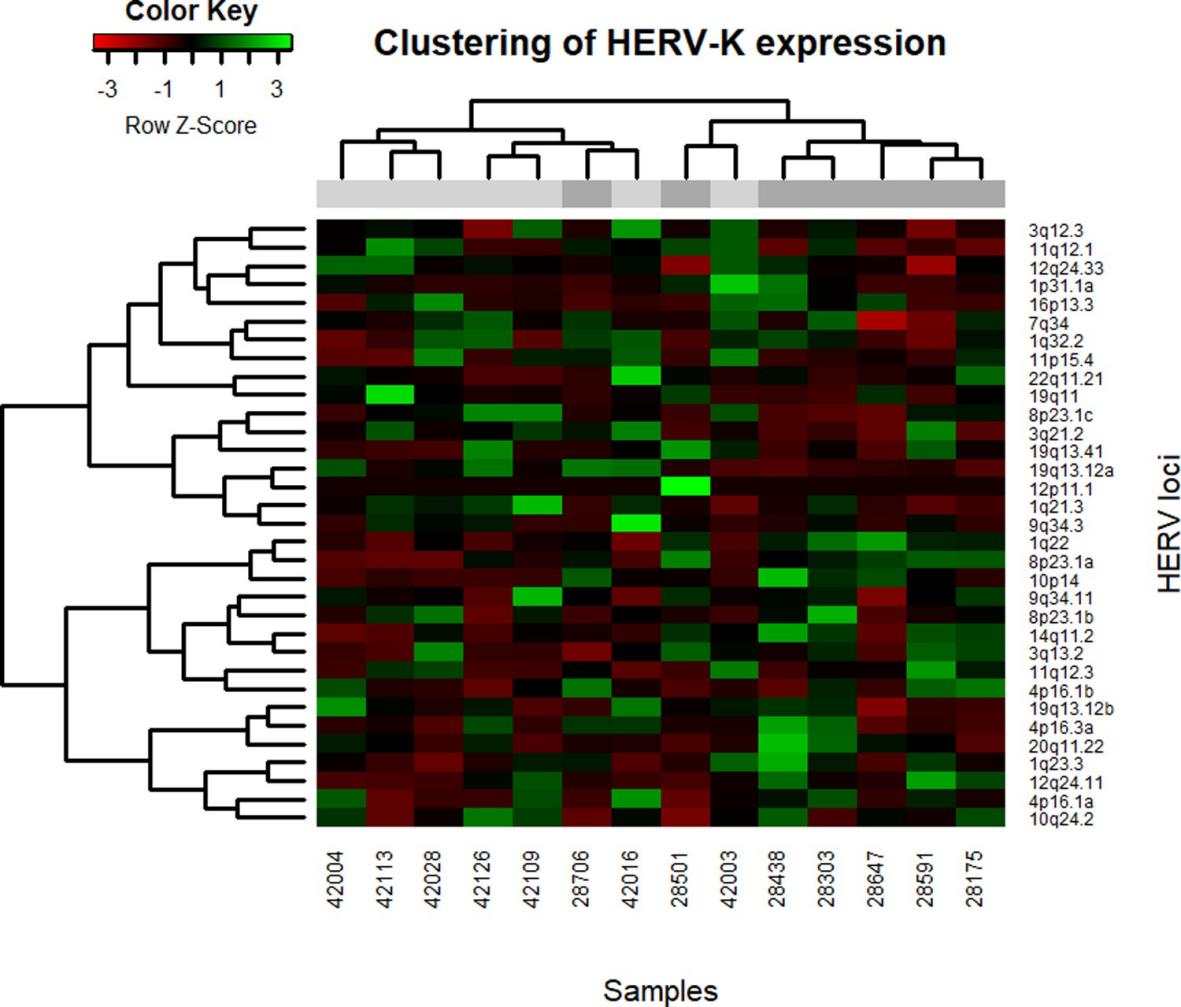
Heatmap of clustered samples and proviruses based on HERV-K (HML-2) normalized read counts. The color green indicates relatively high expression, while red indicates relatively low expression. The grey blocks beneath sample dendrogram indicate age group membership, with light grey for young controls and dark grey for nonagenarians.

**Fig 3:**
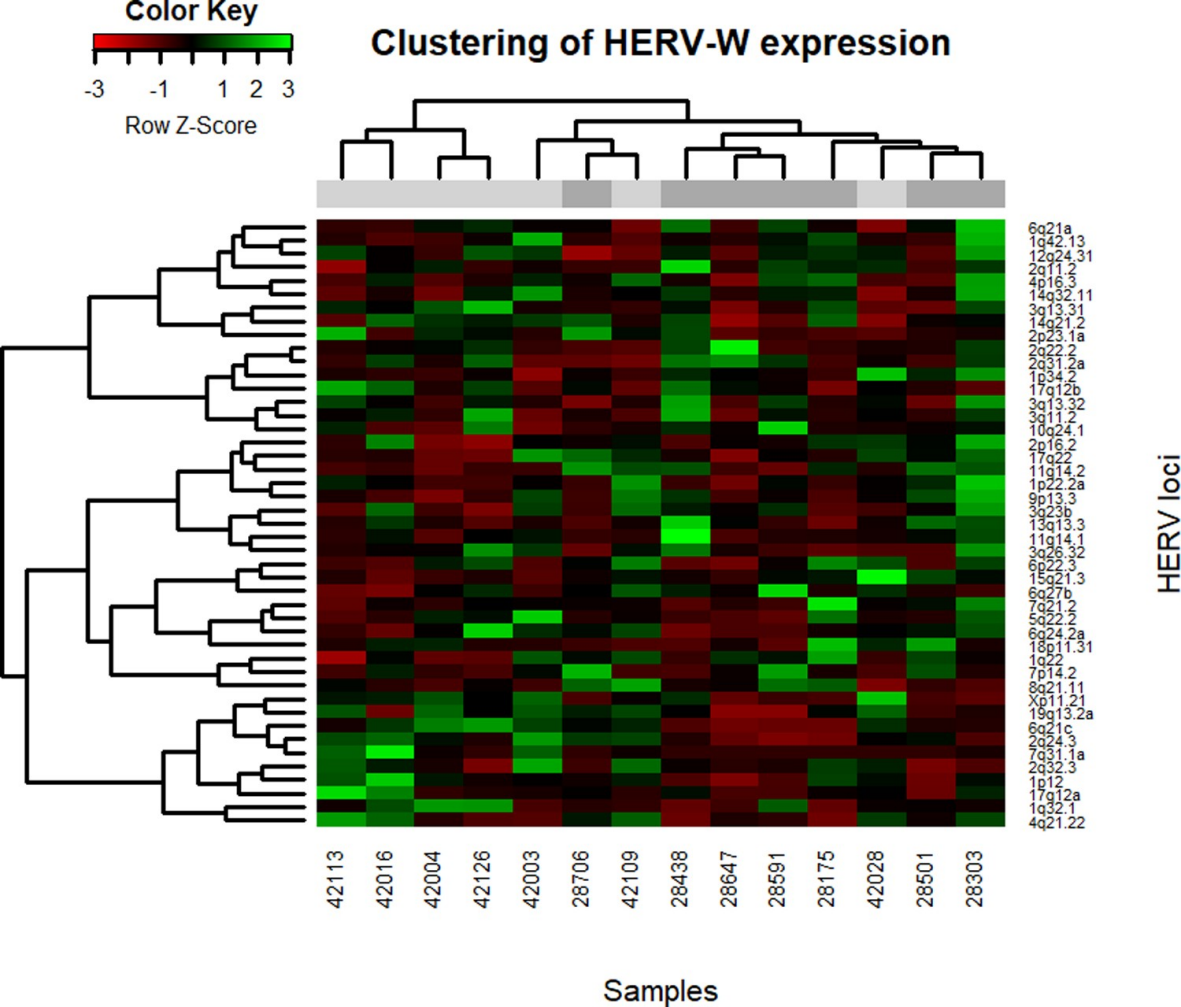
Heatmap of clustered samples and proviruses based on HERV-W normalized read counts. The color green indicates relatively high expression, while red indicates relatively low expression. The grey blocks beneath sample dendrogram indicate age group membership, with light grey for young controls and dark grey for nonagenarians. HERV-W has more proviruses listed, which causes changes to the appearance of the heatmap, in addition to the differences in expression.

Hierarchical clustering of the samples based on normalized provirus read counts was done to investigate expression patterns. Clustering of samples based on HERV-K (HML-2) expression resulted in two groups separated along the age group lines (Fig 1A). There were two deviations from this, with one nonagenarian in the predominantly young sample cluster and one young sample in the nonagenarian cluster. Heatmaps of the clustering of the HERV-K (HML-2) and HERV-W provirus expression levels are shown in Figs 2 and 3, respectively.

Bootstrap resampling of the clustering was done to quantify the certainty of the clustering. Both clusters have an approximately unbiased (AU) p-value of 97, which is the bias corrected percentage of resampling dendrogram variants where the specific cluster was observed. AU p-value of 97 is equivalent to a p-value of 0.03, indicating statistical significance. The same significant clusters resulted even if the significantly differentially expressed 1q22, 10p14 and 12q24.33 were excluded from clustering.

HERV-W expression based clustering of samples resulted in one statistically significant cluster (p-value of 0.04), which contains the same six nonagenarian samples that are grouped together in the HERV-K (HML-2) based clustering (Fig 1B).

## Conclusions

The RNA levels of individual proviruses varied considerably between samples. It was not the case that some individuals would have been more active producers than others, but instead different proviruses seemed to be expressing non-systematically within and between individuals. A total of eight HERV-K proviruses and nine HERV-W proviruses were found to be expressed in all 14 samples and consequently these proviruses were expressed with highest RNA levels. This suggests that some individual proviruses could be less restricted in terms of their expression potential, that is brought by the regulation machinery of the cell. Several proviruses were expressed only in small part of individuals and it is tempting to think that these could be the ones behind potential adverse effects, especially if they would be mainly expressed in nonagenarians, as they are probably silenced for a reason. There were no proviruses that were expressed exclusively in nonagenarians, but for example HERV-K 8p23.1a was expressed in 6 nonagenarians and only in 1 young individual. Furthermore, only 4 aging-associated differentially expressed proviruses were identified (in HERV-K (HML-2) 1q22 and 10p14 having a higher and 12q24.33 a lower expression in the elderly and in HERV-W Xp11.21 a lower expression). Putting all this together, it seems to be the case, that aging has only moderate effect on the expression levels of individual proviruses.

However, the hierarchical clustering of the expression data indicated that the expression profiles of the young and elderly subpopulations were different. The simplest way to achieve this kind of difference would be if, for example, all the proviruses were expressed systematically slightly up or down in one of the groups. This kind of behavior could be attributed to some kind of common regulator that has only one simple mode of action. However, this was not the case, as different proviruses were up- and downregulated equally in the nonagenarians. This requires more complex regulation and is possibly reflecting multilayered epigenetic regulation machinery involving, among other things, DNA methylation and histone modifications, and inducing distinguishable aging-associated expression profile. Due to Spearman correlation based distance metric in the clustering, each provirus has identical weight in the clustering result, regardless of level of expression. Therefore this result would indicate that there are differences between the age groups that are revealed when the proviral expression profiles are examined as a whole. The underlying cause behind observed expression profile difference thus has to affect the expression of many different proviruses. Understanding what causes this difference could increase knowledge of HERV expression associated disease states and of age-related decline. Since the same nonagenarian samples are clustered by both HERV-K (HML-2) and HERV-W expression, this phenomenon may not be limited to these families, and could be present in other HERV families as well. It is noteworthy, that our analysis is only limited to HERV-K (HML-2) and HERV-W families. Previous studies have indicated that upregulation of some other HERV subclasses might also have implications in tumor immunity [26–28]. Therefore, it is possible that these HERVs could contribute to aging more than HERV-K (HML-2) and HERV-W. This remains to be explored in future studies.

There is a general agreement that the expression of HERVs should be under a strict control, i.e. allowing their expression in the germ line but silencing in most somatic cells, where their activity could disrupt normal gene expression or transcript processing. Several of these control mechanisms have been characterized in detail [29, 30]. As human aging is associated with dramatic epigenetic changes, e.g. DNA methylation [31], it is maybe surprising that expression levels between the young and old individuals were not strikingly different. However, it is possible that this epigenetic regulation is responsible for the observed differences and the expression profiles would be due to differential sensitivity of the individual proviruses to these aging-associated epigenetic changes.

The general expression profile of HERV-K (HML-2) in the resting blood cells used here, was dominated by a few loci, i.e. 3q12.3, 19q13.12b and 1q22., resembling the situation in in vitro pre-activated lymphocytes [32], suggesting that the proliferative state of the cells has probably only a minor effect. This far, no similar data in the case of HERV-W is available.

In conclusion, transcriptional regulation of the proviruses belonging to HERV-K (HML-2) and HERV-W families appears to be two-dimensional in the PBMCs; a subset of HERVs are expressed constantly in age-independent manner having only slight aging-associated differences in the expression levels. These differences might be explained by a fine-tuning of transcriptional regulation that is brought by DNA methylation and is known to be heavily altered in aging. On the other hand, proviruses in another subset of HERVs were characterized by total lack of expression in some individuals. This could be the result of some more drastic mode of regulation such as that of H3K4me3, that is also known to be altered in aging [33]. Aging-dependent HERV profile found with clustering might reflect this aspect of regulations and it is also possible that adverse effects of HERVs are driven by those proviruses that undergo more radical transcriptional relaxation or restriction, that is not necessarily seen in the median RNA levels (for example HERV-K 8p23.1a in Fig 2 and HERV-W 11q14.2 in Fig 3).

This finding might have some practical consequences. In the clinical studies demonstrating associations with HERV-expression the expression of only one or a few proviruses have been used as the indicator. In these studies, analysis of the whole HERV profile would help in finding the true pathogenic provirus. Small number of samples is a limitation of this study, and more comprehensive studies with bigger sample populations are needed for confident evaluation of the RNA levels.
